# A Theoretical and Practical Treatise on the Hemorrhoidal Disease

**Published:** 1885-01

**Authors:** 


A THEORETICAL AND PRACTICAL TREATISE ON THE HEMORRHOIDAL DISEASE, giving its history, nature, causes, pathology, diagnosis and treatment, by William Bodenhamer, A. M., M. D., illustrated by two chromo-lithographic plates and thirty-one wood-cuts. New York: William Wood & Co., 56 and 58 LaFayette Place, 1884.

If the reader will spare himself some unnecessary labor, which devolved upon us in the perusal of the first and second chapters, even to a double translation of a passage from Hippocrates, he will be none the less able to appreciate the proper outline of the characteristics of piles contained in the third chapter; the clear representation of its concomitants in the fourth, and the causative influences which operate in producing this disorder contained in the fifth chapter.

Barring the colored plates of the sixth chapter, which fail to impart a correct hue to the surroundings of the parts represented, the delineation of hemorrhoidal tumors is very satisfactory.

The treatment of piles by the author is subject to serious discount, in his discarding the ecraseur and injection with carbolic acid, and it must be inferred that he has shut his eyes to the great advantages obtained by others in resorting to these efficient measures for the removal of hemorrhoidal tumors.

As there were twenty-two pages at the opening of the book without any practical bearing, so there are thirty-three pages at the close, in the form of a bibliographical catalogue, which will have little interest for the active practitioner, or even for the student looking to the proper management of this class of diseases.

With some marked differences from the views of Dr. Kelsey, pertaining to the rectum and anus, the theory and practice of our author are judicious and commendable, the general principles being for the most part correct.

